# Characterization of high pathogenicity avian influenza H5Nx viruses from a wild harbor seal and red foxes in Denmark, 2021 and 2022

**DOI:** 10.1111/irv.13208

**Published:** 2023-10-15

**Authors:** Yuan Liang, Charlotte K. Hjulsager, Tim K. Jensen, Anne Sofie V. Hammer, Maibritt T. Ovesen, Lars E. Larsen

**Affiliations:** ^1^ Department of Veterinary and Animal Sciences University of Copenhagen Frederiksberg Denmark; ^2^ Department of Virus and Microbiological Special Diagnostics Statens Serum Institut Copenhagen Denmark

**Keywords:** Denmark, high pathogenicity avian influenza, influenza A virus, mammals, wild birds

## Abstract

In 2021 and 2022, clade 2.3.4.4b H5Nx high pathogenicity avian influenza viruses were detected in one harbor seal and in one adult and three fox cubs in Denmark. The viruses were closely related to contemporary viruses found in Europe, and some had obtained amino acid substitutions related to mammalian adaptation. Notably, the virus distribution appeared to have been different in the infected fox cubs, as one exclusively tested positive for the presence of HPAIV in the brain and the other two only in the lung. Collectively, these findings stress the need for increased disease surveillance of wild and farmed mammals.

## 1 MAMMALIAN CASES IN DENMARK

**FIGURE 1:**
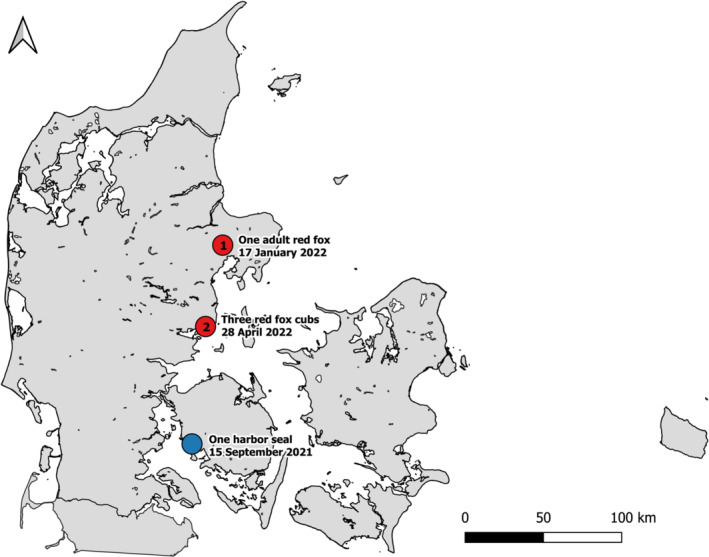
Map of Denmark showing where the HPAIV‐positive harbor seal (blue dot), adult fox (red dot marked with “1”), and three red fox cubs (red dot marked with “2”) were detected. The date of observation is also indicated next to the location site. The harbor seal was found at a beach on Southwestern Funen. The adult fox and fox cubs were all detected on the eastern part of Jutland.

In addition to wild and domestic birds, many mammals have been affected by high pathogenicity avian influenza virus (HPAIV) in recent seasons. In 2021 and 2022, five mammals tested positive for clade 2.3.4.4b H5Nx HPAIVs in Denmark. The first mammalian case was an adult male harbor seal ( *Phoca vitulina* ), found dead in September 2021 on a beach at Southwest Funen (Figure 1). The seal was in a state of progressed decay and was assessed to have been dead for up to 1 week at sea. At necropsy, the seal was found emaciated. The heart and respiratory organs appeared unaffected, whereas the abdominal organs were too decayed for thorough examination. The organs were unsuitable for histology. The organs were also tested for the presence of pathogenic bacteria and morbillivirus as previously described,^1^ in which none tested positive. The presence of clade 2.3.4.4b HPAIV H5N8 was confirmed in the lung of the seal by real‐time RT‐PCR (rRT‐PCR) and sequencing as previously described^2^ (Table 1).

**TABLE 1:** Summary of the virological results and histopathological examinations of the mammals infected with HPAIV.

Mammal	Sample	M‐gene Ct	Virus subtype	Virus name	Histopathology	Immuno‐histochemistry
Harbor seal	Lung	28.98	H5N8	A/harbour seal/Denmark/521–2/2021 (EPI_ISL_10053635)	N/A (postmortem decay)	N/A
Red fox adult	Lung	21.96	H5N1	A/red fox/Denmark/01679–1.01‐2/2022 (EPI_ISL_17447412)	Fibrinous and necrotizing pneumonia	+
Red fox cub 1	Lung				Fibrinous and interstitial pneumonia	−
Red fox cub 1	BRAIN	19.39	H5N1	A/red fox/Denmark/08658–1.01/2022 (EPI_ISL_17447413)	N/A	N/A
Red fox cub 2	Lung	26.50	H5N1	A/red fox/Denmark/08658–2.02‐2/2022 (EPI_ISL_17447414)	Fibrinous pneumonia	+
Red fox cub 2	Brain				‐	−
Red fox cub 3	Lung	26.50	H5N1	A/red fox/Denmark/08658–3.02‐2/2022 (EPI_ISL_17447440)	Fibrinous and interstitial pneumonia	−
Red fox cub 3	BRAIN				−	−

Abbreviations: +, positive staining; −, no lesions or staining; EPI_ISL, virus ID in GISAID EpiFlu sequence database; N/A, not analyzed.

**FIGURE 2:**
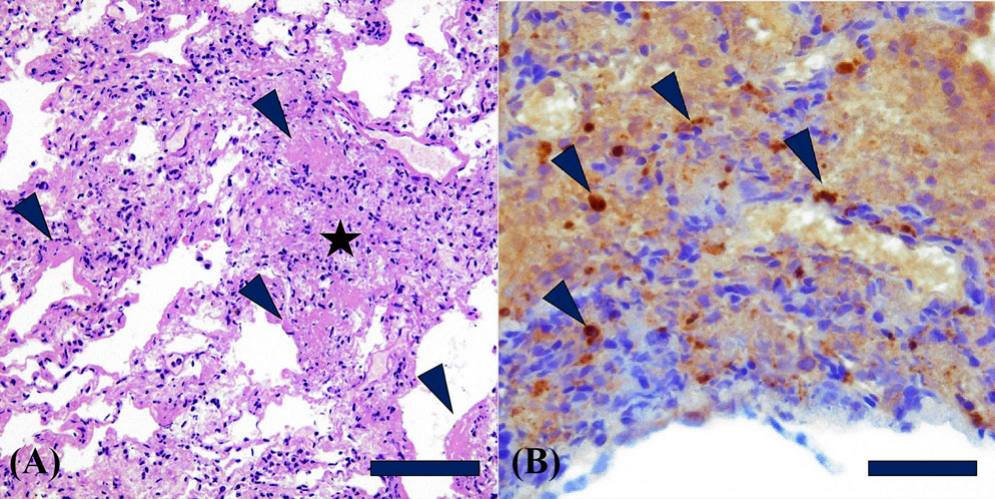
Lung tissue from two red foxes with pneumonia consistent with influenza infection. The lungs tested positive for HPAIV by rRT‐PCR. (A) Adult fox with fibrinous to necrotizing lesions. Clots of fibrin (arrowhead) in alveoli and lining the terminal bronchi intermixed with small necrosis (star). Hematoxylin and eosin, bar 100 μm. (B) Fox cub with non‐suppurative interstitial to fibrinous pneumonia with multiple cells staining positive for influenza virus (dark brown/arrow head) by immunohistochemistry using a mouse monoclonal antibody against influenza A virus NP, bar 50 μm.

In 2022, HPAIV H5N1 was detected in one adult red fox and three red fox cubs ( *Vulpes vulpes* ) by rRT‐PCR and sequencing.^2^ Brain and/or lung tissues from the four foxes were collected for virological and histopathological analyses. The adult fox was found dead on January 17, 2022, close to a fox pit at Djursland in Eastern Jutland, where HPAIV H5N1 was detected in the lung (Table 1). On April 28, 2022, three fox cubs were found dead in another location, 77 km southwest of Djursland, next to a fox pit in Odder municipality. All three fox cubs were observed alive 3 days prior. HPAIV H5N1 was detected by rRT‐PCR in brain tissue from one of the fox cubs and in the lung of the other two cubs. The rRT‐PCR analysis was repeated with the same results. The carcass of a bird, most likely a common scoter ( *Melanitta nigra* ), was found next to the fox cubs. Unfortunately, the carcass was discarded before any samples could be collected. Macroscopic findings at necropsy of the foxes included pulmonary edema and consolidation, and the livers were enlarged with congestion. Pronounced emphysema in the cranial parts of the lung and hepatic steatosis were also detected in the fox cubs. Based on size and weight, the age of the fox cubs was estimated to be 4–5 weeks. The adult fox was in poor body condition, while the body conditions of the fox cubs were within normal range. Pneumonic lesions in all HPAIV‐positive foxes were revealed by histopathology. In the adult fox, the lesions were dominated by fibrinous to necrotizing pneumonia (Figure 2A), while the three fox cubs revealed varying degrees of fibrinous to interstitial pneumonia. In addition, influenza A virus (IAV) nucleoprotein (NP) antigen was demonstrated in the lung of the adult fox and in one of the cubs by immunohistochemistry using a mouse monoclonal antibody against the IAV NP protein as primary antibody (HYB 340‐05, www.ssi.dk/antibodies) (Figure 2B). No significant lesions were detected in other organs (kidney, small intestine, liver, and brain); however, postmortem decay of the organs made the histopathologic evaluation difficult. Additionally, all four foxes were assessed for infection with pathogenic bacteria in the liver and lungs and morbillivirus in lungs, spleen, and for the cubs also in brain.^1^ The adult fox was furthermore tested for Aleutian mink disease virus (AMDV).^3^ No pathogenic bacteria or viruses, other than HPAIV, were identified in any of the foxes. The HPAIV H5N1 positive adult fox and fox cubs were investigated as part of the 2022 wild mammal disease surveillance program in Denmark. In 2022, the presence of IAV was investigated in further five foxes, a European badger and a European polecat that were found dead in nature, as well as in 35 foxes, 2 common raccoons, 1 house marten, 1 wolf, 1 European badger, 1 common raccoon dog, and 1 European polecat that were apparently healthy and killed due to regulation or killed in road traffic collisions. IAV was not detected in any of these mammals.

**FIGURE 3:**
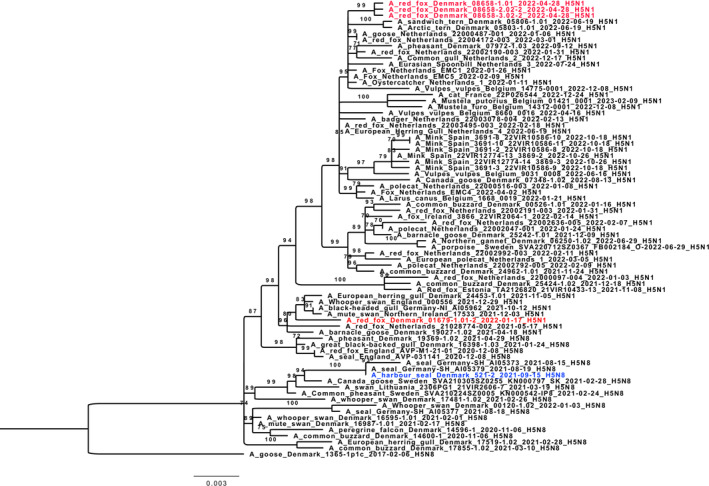
Maximum likelihood phylogenetic tree inferred from hemagglutinin (HA) gene of clade 2.3.4.4b H5Nx HPAIVs. The mammalian viruses found in Denmark were compared to H5 HPAIVs from wild birds and mammals detected in other European countries. Viruses with a high genetic similarity determined by BLAST on GISAID EpiFLu (wwww.gisaid.org) also included in the analysis. HPAIVs detected in Denmark in the same time period and a representative set of H5 sequences of viruses were obtained from the GISAID EpiFLu database and included in the analysis. The trees were estimated using IQ‐Tree version 2.0.3. Only bootstrap values of ≥70 are shown. The scale bar indicates the nucleotide substitutions per site.

**FIGURE A1:**
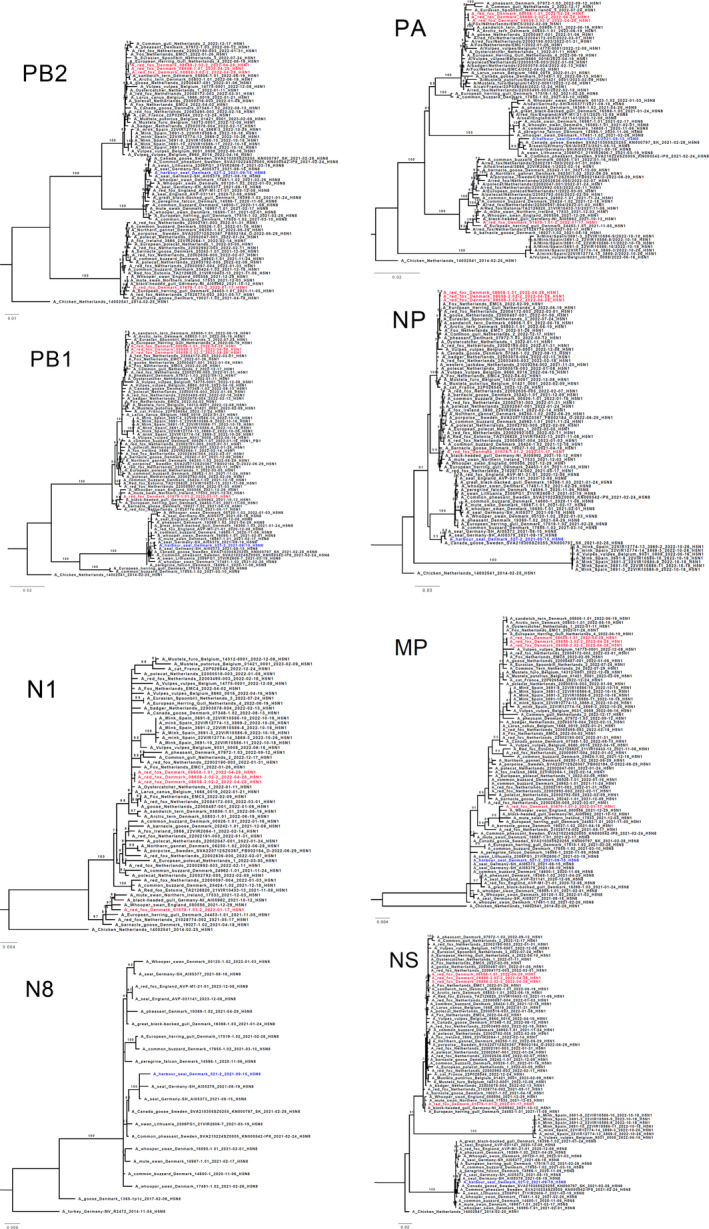
Maximum likelihood phylogenetic tree inferred on the gene segments, polymerase basic 2 (PB2), polymerase basic 1 (PB1), polymerase acidic (PA), nucleoprotein (NP), neuraminidase (NA) subtype N1, NA subtype N8, matrix protein (MP), and non‐structural protein (NS). The mammalian viruses found in Denmark were compared to H5 HPAIVs from wild birds and mammals detected in other European countries. Viruses with a high genetic similarity determined by BLAST on EpiFLu™ GISAID (www.gisaid.org) were also included in the analysis. The trees were estimated using IQ‐Tree version 2.0.3. HPAIVs detected in Denmark in the same period and a representative set of H5 sequences of viruses were obtained from the EpiFLu™ GISAID database and included in the analysis. Only bootstrap values of ≥70 are shown. The scale bar indicates the nucleotide substitutions per site.

The viruses from the seal and all four foxes were full genome sequenced as previously described^4^ and compared to sequences of related and contemporary viruses from the GISAID EpiFlu™ database (http://www.gisaid.org). Full‐genome sequences were generated for the viruses found in red fox cubs 1 and 3, while it was only possible to generate partial genome sequences for the viruses in the harbor seal and red fox cub 2. Phylogenetic analysis identified the viruses to belong to clade 2.3.4.4b of the Goose/Guangdong lineage. The H5N1 virus from the cubs and the adult fox belonged to two different genotypes of clade 2.3.4.4b H5Nx HPAIV, A/duck/Saratov/29‐02/2021‐like and A/Eurasian Wigeon/Netherlands/1/2020‐like, respectively (Figures 3 and A1) (data on the genotyping are not shown). Comparing the available genome sequences, viruses detected in red fox cubs 1 and 2 were 100% identical on amino acid level, whereas they differed to red fox cub 3 by six nucleotides in which four resulted in amino acid substitutions (PB2‐E627K, HA‐R456G [H5‐numbering], NP‐E454K, and NP‐R485G). Viruses from the adult fox and the fox cubs were all closely related to viruses found in contemporary wild birds from Denmark, while the virus from the seal was closest related to a virus found in a seal in the German part of the North Sea in August 2021^5^ (Figures 3 and A1). Analysis of the amino acid sequence revealed substitutions related to adaptation to mammals (Table A1). Notably, the PB2‐E627K substitution was present in the viruses found in the lung of the harbor seal and the lung of one of the fox cubs (A/red fox/Denmark/08658‐3.02‐2/2022) (Table A1). This mutation has been linked to mammalian adaptation and has in recent years been detected in several HPAIVs from infected mammals in Europe.^6^

## 2 DISCUSSION

Here, we describe the first documented mammalian infections with clade 2.3.4.4b H5Nx HPAIVs in Denmark. While there have been recent reports of clade 2.3.4.4 H5N1 HPAIV in several species of wild mammals, most of the affected species have been seals and foxes,^7^ which has also been the case in Denmark. Although other causes of deaths cannot be completely ruled out, we postulate that the most probable explanation was infection with HPAIV. This assumption is strongly supported by the lung lesions consistent with HPAIV infection, the molecular detection of the virus by rRT‐PCR and sequencing, and the detection of IAV antigen in the lungs by immunohistochemistry combined with no other obvious cause of death having been identified. The genetic analyses combined with the epidemiological data indicate that there was likely no transmission of HPAIV between the adult fox and cubs, since the viruses were of two different H5N1 genotypes. The viruses detected in the fox cubs were genetically closely related; however, it is unclear if they were infected by eating the same infected bird or by virus transmission among the cubs. It could also be hypothesized that the fox cubs were infected by their mother although no sick or dead vixen was observed in the surrounding area. Similarly, the analyses performed of the HPAIV‐infected seal also strongly indicated infection by HPAIV to be the cause of death. Interestingly, the HPAIV detected in the Danish harbor seal was most similar to viruses detected in two harbor seals in the German part of the North Sea found dead 1 month earlier.^5^ This could indicate that these seals either had a common source of infection or that there could potentially have been seal‐to‐seal transmission. During a previous outbreak of H10N7 AIV in 2014–2015, there was clear evidence of transmissions among seal populations from both the Baltic Sea and the North Sea.^8^ However, it is important to note that the genome was only partially generated for the HPAIV found in the harbor seal in Denmark.

Pneumonic lesions have been associated with HPAIV infection in other mammals, including ferrets, cats, and dogs,^9^, ^10^, ^11^, ^12^ which are in agreement with the lesions that were detected in the seal and three of the foxes. In this study, no evident signs of lung infection were revealed in the seal. Previous investigations have shown that HPAIV induces central nervous system infections in mammals and report that the viral load is higher in the brain in comparison to the respiratory tract.^5^, ^13^, ^14^ Notably, we were able to detect viral RNA only in the brain of one red fox cub, whereas the remaining fox cubs were only virus positive in the lung. It is uncertain whether the disparities in virus distribution could reflect variation in organ tropism or if it reflects the differences in infection progression, transmission route, and/or dose of exposure. While HPAIV is commonly neurotropic among mammals, our results stress the importance of including multiple organs when testing for infection with HPAIV.

Although AIVs are known to infect many mammalian species, there has been an increasingly extensive mortality among wild mammals associated with HPAIV since 2020.^6^ Multiple spillover events of clade 2.3.4.4b HPAIVs to both marine and terrestrial mammals have been reported across multiple countries. Many of these spillover events have been onto mammalian species that previously had never been affected by HPAIV. The increased number of detections of mammalian HPAIV cases with markers of genetic mammalian adaption is worrisome, as it may lead to a higher risk of human infections and calls for enhanced one health‐based AIV surveillance including both wild and farmed mammals.

## AUTHOR CONTRIBUTIONS

**Yuan Liang:** Conceptualization; formal analysis; investigation; visualization; writing—original draft; writing—review and editing. **Charlotte K. Hjulsager:** Conceptualization; formal analysis; investigation; writing—original draft; writing—review and editing. **Tim K. Jensen:** Conceptualization; formal analysis; investigation; visualization; writing—original draft; writing—review and editing. **Anne Sofie V. Hammer:** Conceptualization; formal analysis; investigation; visualization; writing—original draft; writing—review and editing. **Maibritt T. Ovesen:** Investigation; writing—review and editing. **Lars E. Larsen:** Conceptualization; formal analysis; investigation; writing—original draft; writing—review and editing.

## CONFLICT OF INTEREST STATEMENT

All authors declare that they have no conflict of interest.

### PEER REVIEW

The peer review history for this article is available at https://www.webofscience.com/api/gateway/wos/peer-review/10.1111/irv.13208.

## Data Availability

All sequence data generated and used in this study have been uploaded to the GISAID EpiFlu database (https://www.gisaid.org/). Accession numbers are provided in the table.

## APPENDIX A

**TABLE A1:** Mutations observed in the eight gene segments of the HPAIV viruses found in a seal and foxes in Denmark.

Gene segment	Virus	Mutation	Phenotype	Reference
PB2	A_harbour_seal_Denmark_521‐2_2021 A_red_fox_Denmark_08658‐3.02_2022	E627K	Mammalian adaptation	Manzoor et al.^15^
PB1‐F2	A_red_fox_Denmark_01679‐1.01_2022 A_red_fox_Denmark_08658‐1.01_2022 A_red_fox_Denmark_08658‐2.02‐2_2022 A_red_fox_Denmark_08658‐3.02‐2_2022	N66S^a^	Increases virulence	Schmolke et al.^16^
NP	A_harbour_seal_Denmark_521‐2_2021 A_red_fox_Denmark_01679‐1.01_2022	M105V	Increases virulence in chickens	Tada et al.^17^
NP	A_red_fox_Denmark_08658‐3.02‐2_2022	E454K	Increases replication in human cell line	Danzy et al.^18^
NP	A_red_fox_Denmark_01679‐1.01_2022	Y52H^b^	Increases replication in humans	Pinto et al.^19^
M1	A_harbour_seal_Denmark_521‐2_2021 A_red_fox_Denmark_01679‐1.01_2022 A_red_fox_Denmark_08658‐1.01_2022 A_red_fox_Denmark_08658‐2.02‐2_2022 A_red_fox_Denmark_08658‐3.02‐2_2022		Increases virulence in mice	Fan et al.^20^
M1	A_harbour_seal_Denmark_521‐2_2021 A_red_fox_Denmark_01679‐1.01_2022 A_red_fox_Denmark_08658‐1.01_2022 A_red_fox_Denmark_08658‐2.02‐2_2022 A_red_fox_Denmark_08658‐3.02‐2_2022	T215A^a^	Increases virulence in mice	Fan et al.^20^
NS1	A_harbour_seal_Denmark_521‐2_2021 A_red_fox_Denmark_01679‐1.01_2022 A_red_fox_Denmark_08658‐1.01_2022 A_red_fox_Denmark_08658‐2.02‐2_2022 A_red_fox_Denmark_08658‐3.02‐2_2022	P42S^a^	Increases virulence in mice	Jiao et al.^21^
NS1	A_harbour_seal_Denmark_00521‐2_2023 A_red_fox_Denmark_01679‐1.01_2022 A_red_fox_Denmark_08658‐1.01_2022 A_red_fox_Denmark_08658‐2.02‐2_2022 A_red_fox_Denmark_08658‐3.02‐2_2022	P87S^a^	Host‐specificity marker (S in human, P in avian)	Allen et al.^22^
NS1	A_harbour_seal_Denmark_521‐2_2021 A_red_fox_Denmark_01679‐1.01_2022 A_red_fox_Denmark_08658‐1.01_2022 A_red_fox_Denmark_08658‐2.02‐2_2022 A_red_fox_Denmark_08658‐3.02‐2_2022	103F + 106M^a^	Increased virulence in mice	Spesock et al.^23^

^a^ Present in many or all European H5Nx HPAIVs in 2020/2021 and 2021/2022.

^b^ Present in all A/Eurasian Wigeon/Netherlands/1/2020‐like HPAIVs.
